# Beta-blockers reduce intestinal permeability and early mortality following traumatic brain injury in *Drosophila*

**DOI:** 10.17912/micropub.biology.000461

**Published:** 2021-10-01

**Authors:** Amanda R Scharenbrock, Rebeccah J Katzenberger, Megan C Fischer, Barry Ganetzky, David A Wassarman

**Affiliations:** 1 Department of Medical Genetics, School of Medicine and Public Health, University of Wisconsin-Madison, Madison, WI 53706; 2 Department of Genetics, College of Agricultural and Life Sciences, University of Wisconsin-Madison, Madison, WI 53706

## Abstract

Traumatic brain injury (TBI) frequently leads to non-neurological consequences such as intestinal permeability. The beta-blocker drug labetalol, which inhibits binding of catecholamine neurotransmitters to adrenergic receptors, reduces intestinal permeability in a rat TBI model. Using a *Drosophila melanogaster* TBI model, we previously found a strong positive correlation between intestinal permeability and mortality within 24 hours of TBI in a standard laboratory line (*w^1118^*) and across genetically diverse inbred lines from the Drosophila Genetic Reference Panel (DGRP). Here, we report that feeding injured *w^1118^*flies the beta-blockers labetalol and metoprolol reduced intestinal permeability and mortality. Additionally, metoprolol reduced intestinal permeability when 18 DGRP fly lines were analyzed in aggregate, but neither beta-blocker affected mortality. These data indicate that the mechanism underlying disruption of the intestinal barrier by adrenergic signaling following TBI is conserved between humans and flies and that mortality following TBI in flies is not strictly dependent on disruption of the intestinal barrier. Thus, the fly TBI model is useful for shedding light on the mechanism and consequences of adrenergic signaling between the brain and intestine following TBI in humans.

**Figure 1. Beta-blockers reduce intestinal permeability and early mortality following TBI:**
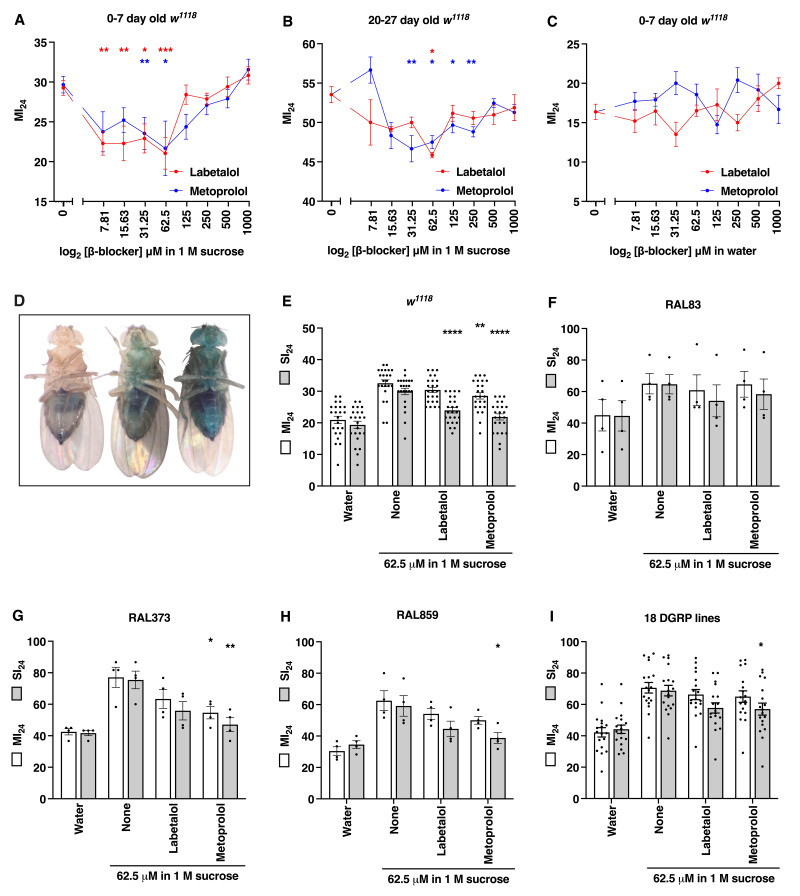
(A-C) The effect of different concentrations of labetalol and metoprolol in (A) 1 M sucrose or (C) water on 0-7 day old *w^1118^* flies and (B) in 1 M sucrose on 20-27 day old *w^1118^* flies. Each dot indicates the average of at least eight biological replicates and error bars indicate the standard error of the mean (SEM). Significance in this panel and others was determined by ordinary one-way ANOVA with Dunnett’s Multiple Comparison test. *p<0.05, **p<0.01, ***p<0.001, and ****p<0.0001. Note that the y-axes are different in panels A-C. (D) Representative images of a non-smurfed fly (left), a smurfed fly at the limit of detection (middle), and an overtly smurfed fly (right). (E-H) The effect of 62.5 μM labetalol and metoprolol in 1 M sucrose on the MI_24_ and SI_24_ of 0-7 day old (E) *w^1118^*, (F) RAL83, (G) RAL373, and (H) RAL859 flies. As controls, flies were fed 1 M sucrose or water. Dots indicate biological replicates, bars indicate the average of at least four biological replicates, and error bars indicate the SEM. (I) The effect of 62.5 μM labetalol and metoprolol in 1 M sucrose on the MI_24_ and SI_24_ of 18 DGRP lines at 0-7 day old (RAL26, RAL83, RAL161, RAL332, RAL352, RAL373, RAL374, RAL382, RAL391, RAL441, RAL491, RAL555, RAL707, RAL761, RAL774, RAL818, RAL859, and RAL907). Dots indicate the average MI_24_ or SI_24_ of four biological replicates for individual DGRP lines, bars indicate the average of the 18 fly lines, and error bars indicate the SEM.

## Description

Traumatic brain injury (TBI) is a substantial public health problem with treatment made difficult by unique neurological sequelae of individual cases (Johnson and Griswold 2017; Pavlovic *et al.* 2019).Pathological processes evolve over time after TBI and are associated with complex changes in neurotransmitter systems (McGuire *et al.* 2019). Relevant neurotransmitters include catecholamines such as norepinephrine, epinephrine, and dopamine that target adrenergic receptors (Jenkins *et al.* 2016). Elevated levels of circulating catecholamines in plasma, in particular epinephrine, at the time of hospital admission after TBI are associated with increased risk of worse functional outcomes and mortality (Woolf *et al.* 1987; Rizoli *et al.* 2017). Furthermore, retrospective and prospective studies show that TBI patients treated with beta-blockers, agents that block binding of epinephrine to its receptor, have a significantly reduced risk of mortality (Cotton *et al.* 2007; Schroeppel *et al.* 2010; Alali *et al.* 2014; Mohseni *et al.* 2015; Ko *et al.* 2016; Khalini *et al.* 2020; Florez-Perdomo *et al.* 2021). Beta-blockers appear to act on trauma-induced signals from the brain, since non-head trauma patients treated with beta-blockers do not have a reduced risk of mortality (Hendrick *et al.* 2016).

Beta-blockers may elicit beneficial effects in TBI by reducing intestinal permeability, as indicated by a study of the beta-blocker labetalol in a rat TBI model (Lang *et al.* 2015). In mammals, bidirectional signaling between the brain and intestine, more generally known as the brain-gut axis, plays a significant role in TBI (Pimentel *et al.* 2012; Al Omran and Aziz 2014; Katzenberger *et al.* 2015c; Mittal *et al.* 2017; Weaver *et al.* 2021). Direct mechanical damage to the brain in rodent TBI models causes disruption of the intestinal barrier, and in the first few weeks after injury, TBI patients frequently have reduced intestinal contractile activity and absorption that can lead to intestinal permeability (Faries *et al.* 1998; Hang *et al.* 2003; Feighery *et al.* 2008; Jin *et al.* 2008; Bansal *et al.* 2009; Bansal *et al.* 2010; Ma *et al.* 2017; Pan *et al.* 2019). Despite evidence that beta-blockers attenuate functional deficits after TBI, more research is needed to understand the underlying mechanisms as well as potentially confounding effects of diverse genetic and environmental factors (Heffernan *et al.* 2010; Osier *et al.* 2016).

To study the effect of genetic and environmental factors on TBI outcomes, we developed a *Drosophila melanogaster* model of closed-head TBI (Katzenberger *et al.* 2013, 2015b). The fly model uses a spring-based, High-Impact Trauma (HIT) device to inflict TBI. Injuries inflicted by the HIT device lead to intestinal permeability and early mortality, suggesting that secondary injury mechanisms are conserved between humans and flies (Katzenberger *et al.* 2013, 2015a, 2016). Our measure of early mortality is the Mortality Index at 24 h (MI_24_), which is the percent mortality of injured flies minus the percent mortality of uninjured flies within 24 h. The MI_24_ is affected by diet following TBI. For example, the MI_24_ is lower for flies fed water versus high-carbohydrate diets following TBI (Katzenberger *et al.* 2015a, 2016). Our measure of intestinal permeability is the Smurfing Index at 24 h (SI_24_), which is the percent of injured flies that smurf minus the percent of uninjured flies that smurf within 24 h. In the Smurf assay, flies are fed a nonabsorbable blue dye prior to the injury. If the intestinal barrier is intact following the injury, the dye remains in the digestive tract, but if the intestinal barrier is disrupted, the dye crosses the barrier into the circulatory fluid (i.e., hemolymph) and disperses throughout the body in a process referred to as ‘smurfing’ because it results in a blue body akin to Smurf cartoon characters (Rera *et al.* 2012; Martins *et al.* 2018). The HIT device does not deliver head-specific injuries, but a crushing injury to the head is sufficient to cause flies to smurf, suggesting that intestinal barrier dysfunction following injuries from the HIT device is due to brain injuries (Katzenberger *et al.* 2015a).

Our prior analyses of genetically diverse inbred fly lines from the Drosophila Genetic Reference Panel (DGRP) revealed that the SI_24_ shows near perfect correlation with the MI_24_, that is, almost every fly that smurfs dies within 24 h, whereas very few flies that do not smurf die within 24 h (Katzenberger *et al.* 2015a). These data suggest that intestinal permeability is closely associated with early mortality in the fly TBI model. Disruption of the intestinal barrier and early mortality via adrenergic signaling is a possibility in the fly TBI model as well because flies synthesize the catecholamines tyramine and octopamine, which are structurally similar to epinephrine (Roeder, 2005), and signaling through tyramine and octopamine receptors modulates brain-wide states such as arousal as well as behaviors such as aggression (Hardie *et al.* 2007; Zhou *et al.* 2008; Busch *et al.* 2009; Andrews *et al.* 2014; Watanabe *et al.* 2017).

To investigate potential roles of adrenergic signaling in early mortality following TBI, we fed 0-7 day old *w^1118^* flies the beta-blocker labetalol or metoprolol at concentrations ranging from 7.81 μM to 1000 μM in 1 M sucrose over the 24 h following TBI and measured the MI_24_. At 62.5 μM, both beta-blockers caused a significant reduction in the MI_24_ (*p*<0.05) (Fig. 1A). A similar beneficial effect of beta-blockers was observed for 20-27 day old *w^1118^* flies that had a higher MI_24_ (*p*<0.05) (Fig. 1B). As is the case for many other compounds tested for efficacy in mammalian TBI models, both labetalol and metoprolol showed U-shaped dose-responses, indicating that too much or too little adrenergic signaling enhances early mortality following TBI (Calabrese *et al.* 2008). In contrast, none of the beta-blocker concentrations in water significantly reduced the MI_24_ when fed to 0-7 day old *w^1118^* flies that had a lower MI_24_ (Fig. 1C). Taken together, these data indicate that adrenergic signaling triggers secondary injuries that promote mortality following TBI in both younger and older flies. Furthermore, different effects of beta-blockers delivered in sucrose versus water suggest that adrenergic signaling enhances carbohydrate-mediated secondary injuries.

To investigate whether adrenergic signaling mediates intestinal permeability following TBI and if so, whether modification of intestinal permeability affects early mortality, we fed 0-7 day old *w^1118^* flies labetalol or metoprolol at 62.5 μM in 1 M sucrose over the 24 h following TBI and measured the SI_24_ and MI_24_. Figure 1D shows the range of blue body coloration that was scored as positive for smurfing. Both beta-blockers significantly reduced the SI_24_ (p<0.001), but in contrast to data in panel A, only metoprolol reduced the MI_24_ (*p*<0.01) (Fig. 1E). Additionally, *w^1118 ^*flies fed water had similar SI_24_ and MI_24_ values that were substantially lower than those of *w^1118 ^*flies fed 1 M sucrose, demonstrating that even when the SI_24_ and MI_24_ are reduced they remain similar when adrenergic signaling is intact. We repeated the experiment with 18 randomly selected lines from the DGRP. Representative data for three lines (RAL83, RAL373, and RAL859) are shown in panels F-H, and collective data for the 18 DGRP lines are shown in panel I. Neither beta-blocker affected the SI_24_ or MI_24_ of RAL83 flies, but metoprolol significantly reduced the SI_24_ (*p*<0.01) and the MI_24_ (p<0.05) of RAL373 flies and metoprolol significantly reduced the SI_24_ (*p*<0.05) of RAL859 flies (Figs. 1F-H). Analysis of the 18 DGRP lines in aggregate showed that metoprolol significantly reduced the SI_24_ (*p*<0.05), but neither beta-blocker affected the MI_24_ (Fig. 1I). Stronger effects of beta-blockers in DGRP fly lines may have been observed if beta-blocker doses were optimized for each line, as they were for *w^1118^* flies. Even so, among flies of different genotype, beta-blockers reduced intestinal permeability to a greater extent than early mortality.

These data indicate that following TBI in flies, adrenergic signaling mediates intestinal permeability and early mortality. Furthermore, since beta-blockers reduced intestinal permeability without affecting early mortality in some fly lines, early mortality is not strictly dependent upon intestinal permeability. However, the disconnect between intestinal permeability and early mortality may be due to the inability of the Smurf assay to detect low amounts of intestinal permeability that could be sufficient to cause mortality. Nevertheless, this study demonstrates that in flies, as in mammals, adrenergic signaling triggers intestinal permeability following TBI. As a result, the fly TBI model can be used to investigate the mechanism underlying TBI-induced adrenergic signaling between the brain and intestine and the influence of genetic and environmental factors on the mechanism.

## Methods

***Fly lines and culturing***

Flies were maintained in humidified incubators at 25°C in vials containing cornmeal molasses food (Katzenberger *et al.* 2015). DGRP lines were obtained from the Bloomington Stock Center, and *w^1118^* flies were obtained from Dr. Gerald Rubin’s lab (University of California-Berkeley) and maintained for 25 years. DGRP lines used in the study included RAL26 (DGRP-26/FBsn0000007), RAL83 (DGRP-83/FBsn0000021), RAL161 (DGRP-161/FBsn0000036), RAL332 (DGRP-332/FBsn0000072), RAL352 (DGRP-352/FBsn0000079), RAL373 (DGRP-373/FBsn0000091), RAL374 (DGRP-374/FBsn0000092), RAL382 (DGRP-382/FBsn0000099), RAL391 (DGRP-391/FBsn0000104), RAL441 (DGRP-441/FBsn0000117), RAL491 (DGRP-491/FBsn0000122), RAL555 (DGRP-555/FBsn0000134), RAL707 (DGRP-707/FBsn0000146), RAL761 (DGRP-761/FBsn0000159), RAL774 (DGRP-774/FBsn0000162), RAL818 (DGRP-818/FBsn0000177), RAL859 (DGRP-859/FBsn0000191), and RAL907 (DGRP-907/FBsn0000202).

***Treatment with beta-blockers***

Stock solutions of 1 mM labetalol and metoprolol (Sigma, St. Louis, MO) were prepared in 1 M sucrose (Sigma) or water and serially diluted 2-fold in 1 M sucrose or water to 7.81 μM. Solutions of labetalol and metoprolol as well as 1 M sucrose and water were fed to flies by placing 200 μl on a filter paper disc at the bottom of a vial.

***MI_24_ and SI_24_ assays***

MI_24_ values were determined as described in Katzenberger *et al.* 2013 and 2015b. SI_24_ values were determined as described in Katzenberger *et al.* 2015a, based on the Smurf assay described in Rera *et al.* 2012 and Martins *et al.* 2018. In panels A-C and E, TBI was inflicted by four strikes from HIT device #1 with 5 min between strikes, and in panels D and F-I, TBI was inflicted by three strikes from HIT device #9 with 5 min between strikes. In each biological replicate, a vial contained 60 flies (approximately 30 males and 30 females).

## References

[R1] Alali AS, McCredie VA, Golan E, Shah PS, Nathens AB (2014). Beta blockers for acute traumatic brain injury: a systematic review and meta-analysis.. Neurocrit Care.

[R2] Al Omran Y, Aziz Q (2014). The brain-gut axis in health and disease.. Adv Exp Med Biol.

[R3] Andrews JC, Fernández MP, Yu Q, Leary GP, Leung AK, Kavanaugh MP, Kravitz EA, Certel SJ (2014). Octopamine neuromodulation regulates Gr32a-linked aggression and courtship pathways in Drosophila males.. PLoS Genet.

[R4] Bansal V, Costantini T, Kroll L, Peterson C, Loomis W, Eliceiri B, Baird A, Wolf P, Coimbra R (2009). Traumatic brain injury and intestinal dysfunction: uncovering the neuro-enteric axis.. J Neurotrauma.

[R5] Bansal V, Costantini T, Ryu SY, Peterson C, Loomis W, Putnam J, Elicieri B, Baird A, Coimbra R (2010). Stimulating the central nervous system to prevent intestinal dysfunction after traumatic brain injury.. J Trauma.

[R6] Busch S, Selcho M, Ito K, Tanimoto H (2009). A map of octopaminergic neurons in the Drosophila brain.. J Comp Neurol.

[R7] Calabrese EJ (2008). Drug therapies for stroke and traumatic brain injury often display U-shaped dose responses: occurrence, mechanisms, and clinical implications.. Crit Rev Toxicol.

[R8] Cotton BA, Snodgrass KB, Fleming SB, Carpenter RO, Kemp CD, Arbogast PG, Morris JA Jr (2007). Beta-blocker exposure is associated with improved survival after severe traumatic brain injury.. J Trauma.

[R9] Faries PL, Simon RJ, Martella AT, Lee MJ, Machiedo GW (1998). Intestinal permeability correlates with severity of injury in trauma patients.. J Trauma.

[R10] Feighery L, Smyth A, Keely S, Baird AW, O'Connor WT, Callanan JJ, Brayden DJ (2008). Increased intestinal permeability in rats subjected to traumatic frontal lobe percussion brain injury.. J Trauma.

[R11] Florez-Perdomo WA, Laiseca Torres EF, Serrato SA, Janjua T, Joaquim AF, Moscote-Salazar LR (2021). A Systematic Review and Meta-Analysis on Effect of Beta-Blockers in Severe Traumatic Brain Injury.. Neurol Res.

[R12] Hang CH, Shi JX, Li JS, Wu W, Yin HX (2003). Alterations of intestinal mucosa structure and barrier function following traumatic brain injury in rats.. World J Gastroenterol.

[R13] Hardie SL, Zhang JX, Hirsh J (2007). Trace amines differentially regulate adult locomotor activity, cocaine sensitivity, and female fertility in Drosophila melanogaster.. Dev Neurobiol.

[R14] Heffernan DS, Inaba K, Arbabi S, Cotton BA (2010). Sympathetic hyperactivity after traumatic brain injury and the role of beta-blocker therapy.. J Trauma.

[R15] Hendrick LE, Schroeppel TJ, Sharpe JP, Alsbrook D, Magnotti LJ, Weinberg JA, Johnson BP, Lewis RH, Clement LP, Croce MA, Fabian TC (2016). Impact of Beta-Blockers on Nonhead Injured Trauma Patients.. Am Surg.

[R16] Jenkins PO, Mehta MA, Sharp DJ (2016). Catecholamines and cognition after traumatic brain injury.. Brain.

[R17] Jin W, Wang H, Ji Y, Hu Q, Yan W, Chen G, Yin H (2008). Increased intestinal inflammatory response and gut barrier dysfunction in Nrf2-deficient mice after traumatic brain injury.. Cytokine.

[R18] Johnson WD, Griswold DP (2017). Traumatic brain injury: a global challenge.. Lancet Neurol.

[R19] Katzenberger RJ, Loewen CA, Wassarman DR, Petersen AJ, Ganetzky B, Wassarman DA (2013). A Drosophila model of closed head traumatic brain injury.. Proc Natl Acad Sci U S A.

[R20] Katzenberger RJ, Chtarbanova S, Rimkus SA, Fischer JA, Kaur G, Seppala JM, Swanson LC, Zajac JE, Ganetzky B, Wassarman DA (2015). Death following traumatic brain injury in Drosophila is associated with intestinal barrier dysfunction.. Elife.

[R21] Katzenberger RJ, Loewen CA, Bockstruck RT, Woods MA, Ganetzky B, Wassarman DA (2015). A Method to Inflict Closed Head Traumatic Brain Injury in Drosophila.. J Vis Exp.

[R22] Katzenberger RJ, Ganetzky B, Wassarman DA (2015). The gut reaction to traumatic brain injury.. Fly (Austin).

[R23] Katzenberger RJ, Ganetzky B, Wassarman DA (2016). Age and Diet Affect Genetically Separable Secondary Injuries that Cause Acute Mortality Following Traumatic Brain Injury in Drosophila.. G3 (Bethesda).

[R24] Khalili H, Ahl R, Paydar S, Sjolin G, Cao Y, Abdolrahimzadeh Fard H, Niakan A, Hanna K, Joseph B, Mohseni S (2020). Beta-Blocker Therapy in Severe Traumatic Brain Injury: A Prospective Randomized Controlled Trial.. World J Surg.

[R25] Ko A, Harada MY, Barmparas G, Thomsen GM, Alban RF, Bloom MB, Chung R, Melo N, Margulies DR, Ley EJ (2016). Early propranolol after traumatic brain injury is associated with lower mortality.. J Trauma Acute Care Surg.

[R26] Lang Y, Fu F, Sun D, Xi C, Chen F (2015). Labetalol Prevents Intestinal Dysfunction Induced by Traumatic Brain Injury.. PLoS One.

[R27] Mackay TF, Richards S, Stone EA, Barbadilla A, Ayroles JF, Zhu D, Casillas S, Han Y, Magwire MM, Cridland JM, Richardson MF, Anholt RR, Barrón M, Bess C, Blankenburg KP, Carbone MA, Castellano D, Chaboub L, Duncan L, Harris Z, Javaid M, Jayaseelan JC, Jhangiani SN, Jordan KW, Lara F, Lawrence F, Lee SL, Librado P, Linheiro RS, Lyman RF, Mackey AJ, Munidasa M, Muzny DM, Nazareth L, Newsham I, Perales L, Pu LL, Qu C, Ràmia M, Reid JG, Rollmann SM, Rozas J, Saada N, Turlapati L, Worley KC, Wu YQ, Yamamoto A, Zhu Y, Bergman CM, Thornton KR, Mittelman D, Gibbs RA (2012). The Drosophila melanogaster Genetic Reference Panel.. Nature.

[R28] Martins RR, McCracken AW, Simons MJP, Henriques CM, Rera M (2018). How to Catch a Smurf? - Ageing and Beyond… *In vivo* Assessment of Intestinal Permeability in Multiple Model Organisms.. Bio Protoc.

[R29] McGuire JL, Ngwenya LB, McCullumsmith RE (2018). Neurotransmitter changes after traumatic brain injury: an update for new treatment strategies.. Mol Psychiatry.

[R30] Mittal R, Debs LH, Patel AP, Nguyen D, Patel K, O'Connor G, Grati M, Mittal J, Yan D, Eshraghi AA, Deo SK, Daunert S, Liu XZ (2017). Neurotransmitters: The Critical Modulators Regulating Gut-Brain Axis.. J Cell Physiol.

[R31] Mohseni S, Talving P, Thelin EP, Wallin G, Ljungqvist O, Riddez L (2015). The Effect of β-blockade on Survival After Isolated Severe Traumatic Brain Injury.. World J Surg.

[R32] Osier ND, Dixon CE (2015). Catecholaminergic based therapies for functional recovery after TBI.. Brain Res.

[R33] Pan P, Song Y, Du X, Bai L, Hua X, Xiao Y, Yu X (2019). Intestinal barrier dysfunction following traumatic brain injury.. Neurol Sci.

[R34] Pavlovic D, Pekic S, Stojanovic M, Popovic V (2019). Traumatic brain injury: neuropathological, neurocognitive and neurobehavioral sequelae.. Pituitary.

[R35] Pimentel GD, Micheletti TO, Pace F, Rosa JC, Santos RV, Lira FS (2012). Gut-central nervous system axis is a target for nutritional therapies.. Nutr J.

[R36] Rera M, Clark RI, Walker DW (2012). Intestinal barrier dysfunction links metabolic and inflammatory markers of aging to death in Drosophila.. Proc Natl Acad Sci U S A.

[R37] Rizoli SB, Jaja BN, Di Battista AP, Rhind SG, Neto AC, da Costa L, Inaba K, da Luz LT, Nascimento B, Perez A, Baker AJ, de Oliveira Manoel AL (2017). Catecholamines as outcome markers in isolated traumatic brain injury: the COMA-TBI study.. Crit Care.

[R38] Roeder T (2005). Tyramine and octopamine: ruling behavior and metabolism.. Annu Rev Entomol.

[R39] Schroeppel TJ, Fischer PE, Zarzaur BL, Magnotti LJ, Clement LP, Fabian TC, Croce MA (2010). Beta-adrenergic blockade and traumatic brain injury: protective?. J Trauma.

[R40] Watanabe K, Chiu H, Pfeiffer BD, Wong AM, Hoopfer ED, Rubin GM, Anderson DJ (2017). A Circuit Node that Integrates Convergent Input from Neuromodulatory and Social Behavior-Promoting Neurons to Control Aggression in Drosophila.. Neuron.

[R41] Woolf PD, Hamill RW, Lee LA, Cox C, McDonald JV (1987). The predictive value of catecholamines in assessing outcome in traumatic brain injury.. J Neurosurg.

[R42] Zhou C, Rao Y, Rao Y (2008). A subset of octopaminergic neurons are important for Drosophila aggression.. Nat Neurosci.

